# Supplementary material for: Tumor-infiltrating immature innate lymphoid cells in colorectal cancer are biased toward ILC1/tissue-resident NK cell differentiation
Source: Nat Commun. 2026 Mar 27;17:3035. doi: 10.1038/s41467-026-71085-9 (PMC13035902; doi:10.1038/s41467-026-71085-9)
Supplement: Supplementary file 1 — Supplementary Information [file 41467_2026_71085_MOESM1_ESM.pdf]

Supplementary Information for

Tumor-infiltrating immature innate lymphoid cells in colorectal cancer are biased toward ILC1/tissue-resident NK cell differentiation

Anne Marchalot, Malin Ljunggren, Christopher Stamper, Whitney Weigel, Christopher Andrew Tibbitt, Isabel Meninger, Ram Vinay Pandey, Miriam Franklin, John W Bassett, Lorenz Wirth, Colorectal Study Group, Ulrik Lindforss, Gabriella Jansson-Palmer, Caroline Nordenvall, Jenny Mjösberg*

*Corresponding Author

This PDF file includes :

Supplementary Figures 1-13

Supplementary Table 1

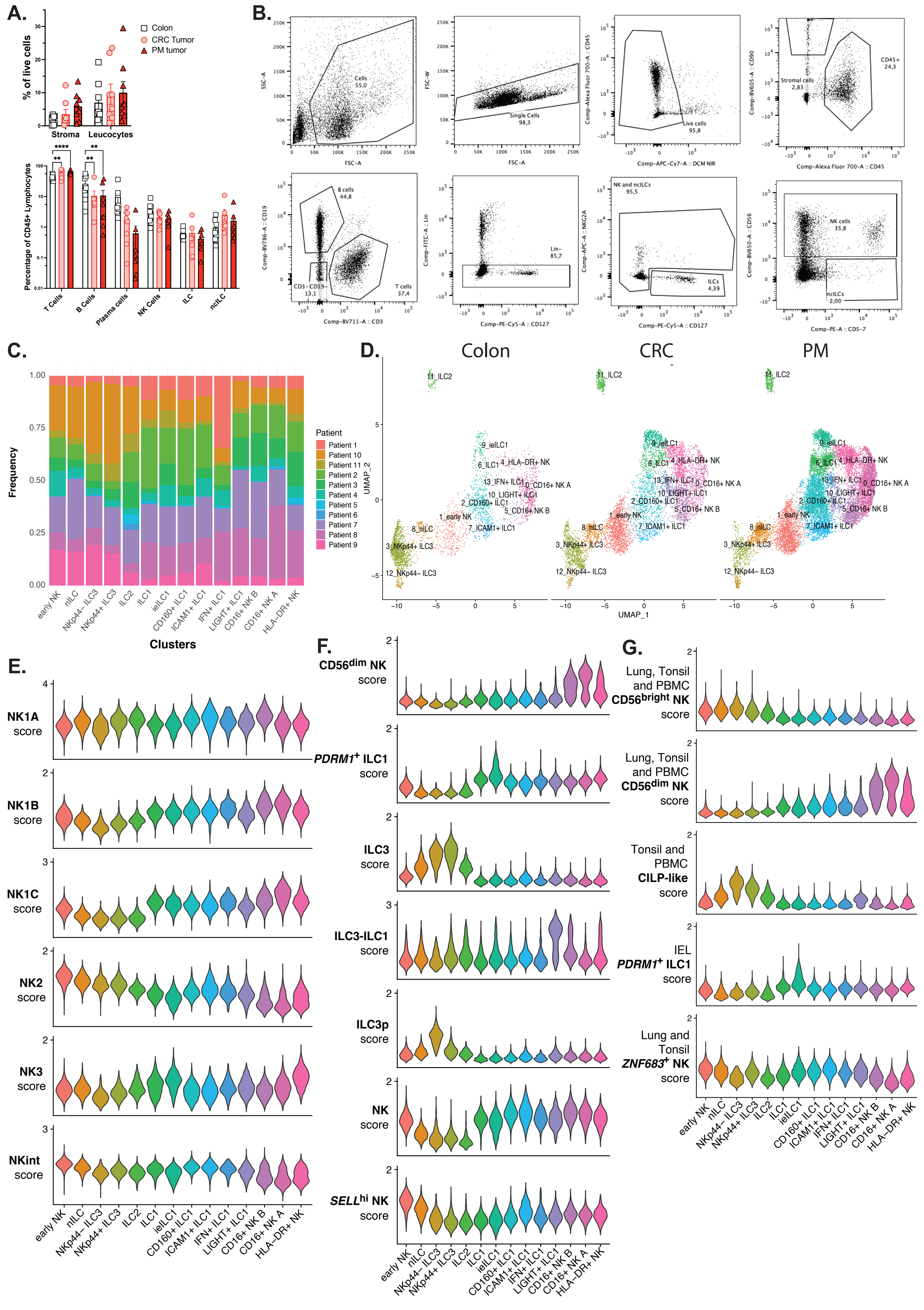
A.
B.
C.
D.
E.
F.
G.
% of live cells
Colon
CRC Tumor
PM tumor
Stroma
Leucocytes
Percentage of CD45+ Lymphocytes
T Cells
B Cells
Plasma cells
NK Cells
ILC
ncILC
Cells 55,0
Single Cells 98,3
Live cells 95,8
Stromal cells 2,83
CD45+ 24,3
B cells 44,8
CD3- CD19- 13,1
T cells 37,4
Lin- 85,7
NK and ncILCs 95,5
ILCs 4,39
NK cells 35,8
ncILCs 2,00
Frequency
Patient
Patient 1
Patient 10
Patient 11
Patient 2
Patient 3
Patient 4
Patient 5
Patient 6
Patient 7
Patient 8
Patient 9
Clusters
Colon
CRC
PM
UMAP_1
UMAP_2
NK1A score
NK1B score
NK1C score
NK2 score
NK3 score
NKint score
CD56dim NK score
PDRM1+ ILC1 score
ILC3 score
ILC3-ILC1 score
ILC3p score
NK score
SELLhi NK score
Lung, Tonsil and PBMC CD56bright NK score
Lung, Tonsil and PBMC CD56dim NK score
Tonsil and PBMC CILP-like score
IEL PDRM1+ ILC1 score
Lung and Tonsil ZNF683+ NK score
early NK
nILC
NKp44- ILC3
NKp44+ ILC3
ILC2
ILC1
ielLC1
CD160+ ILC1
ICAM1+ ILC1
IFN+ ILC1
LIGHT+ ILC1
CD16+ NK B
CD16+ NK A
HLA-DR+ NK

Supplementary Figure 1. Characterization of ILCs in unaffected colon, primary CRC and PM tumors (A) Bar plots representing proportions of each cell type across the colon (white square), CRC tumor (light red circle) and PM tumor (red triangle) as assessed by flow cytometry during FACS. Source data are provided as a Source Data file. Two-way ANOVA with Tukey's post-hoc multiple comparisons test, data shown as mean ± SEM, padj ** < 0.01, **** < 0.0001 **(B)** Flow cytometry sorting gating strategy of NKG2A^{-} CD127^{+} ILCs, CD56^{+} NK cells and CD56^{-} CD7^{+} non-conventional ILCs used for single-cell RNA sequencing analysis, one representative donor shown for a colon sample. **(C)** Stacked barplot of the frequency of cells from each patient in the different scRNAseq clusters. **(D)** UMAP colored by clusters and split between the three tissues **(E)** Module scoring of cluster signatures from Rebuffet et al. **(F)** Module scoring of cluster signatures from Jaeger et al. **(G)** Module scoring of clusters signatures from Jaeger et al. Data are from a total of 19 tissue samples from 11 patients analyzed in 11 independent experiments (one patient per experiment). nILC = naive Innate Lymphoid Cells, ieILC1 = intraepithelial ILC1, ncILC = non conventional ILC, NK = Natural Killer, CRC = Colorectal Cancer, PM = Peritoneal Metastasis.

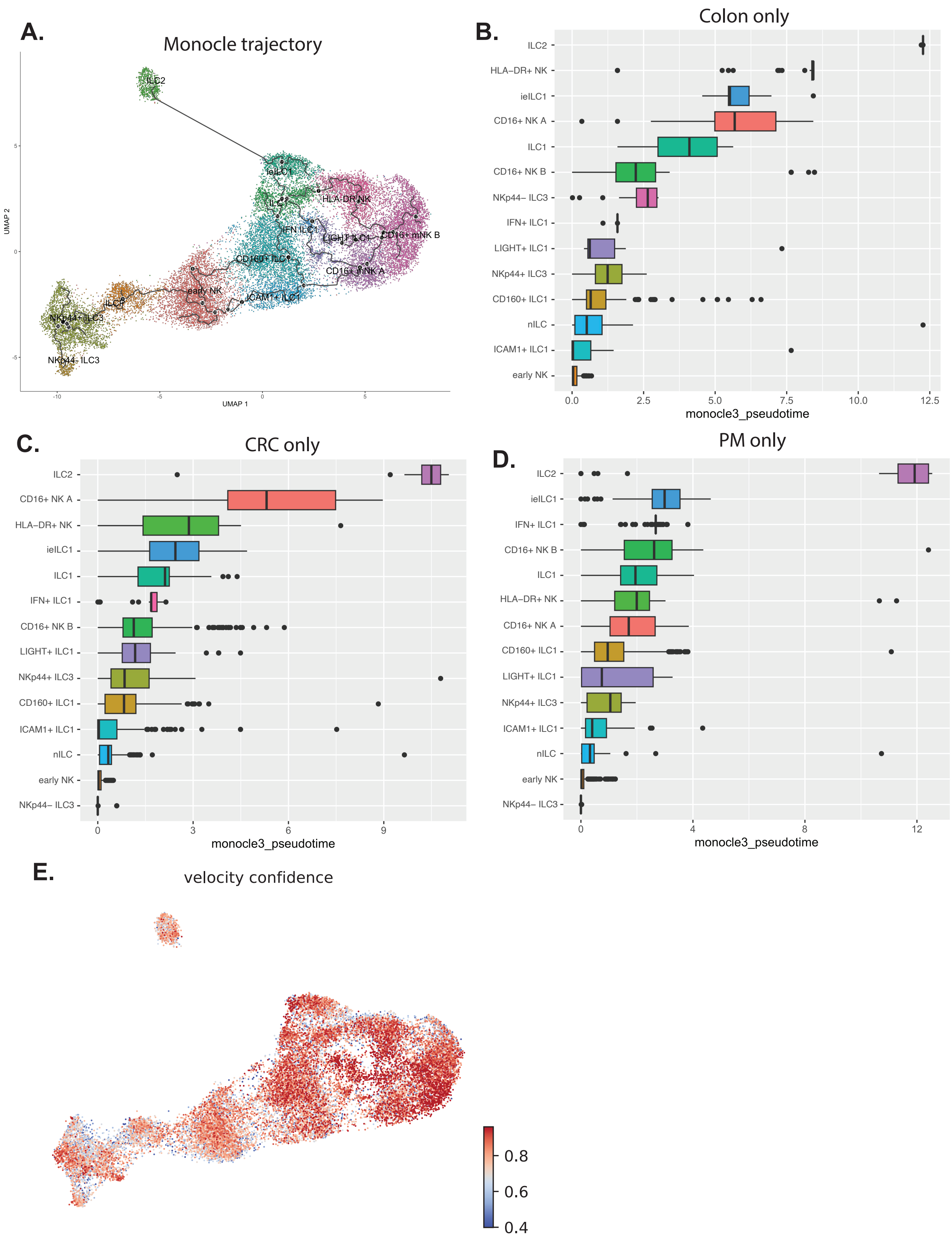
A.
Monocle trajectory
ILC2
ieILC1
ILC1
HLA-DR NK
IFN ILC1
LIGHT ILC1
CD16+ mNK B
CD160+ ILC1
CD16+ mNK A
early NK
ICAM1+ ILC1
ILCP
NKp44+ ILC3
NKp44- ILC3
UMAP 1
UMAP 2
B.
Colon only
ILC2
HLA–DR+ NK
ieILC1
CD16+ NK A
ILC1
CD16+ NK B
NKp44– ILC3
IFN+ ILC1
LIGHT+ ILC1
NKp44+ ILC3
CD160+ ILC1
nILC
ICAM1+ ILC1
early NK
0.0
2.5
5.0
7.5
10.0
12.5
monocle3_pseudotime
C.
CRC only
ILC2
CD16+ NK A
HLA–DR+ NK
ieILC1
ILC1
IFN+ ILC1
CD16+ NK B
LIGHT+ ILC1
NKp44+ ILC3
CD160+ ILC1
ICAM1+ ILC1
nILC
early NK
NKp44– ILC3
0
3
6
9
monocle3_pseudotime
D.
PM only
ILC2
ieILC1
IFN+ ILC1
CD16+ NK B
ILC1
HLA–DR+ NK
CD16+ NK A
CD160+ ILC1
LIGHT+ ILC1
NKp44+ ILC3
ICAM1+ ILC1
nILC
early NK
NKp44– ILC3
0
4
8
12
monocle3_pseudotime
E.
velocity confidence
0.8
0.6
0.4

Supplementary Figure 2. Colon and intratumoral ILCs contain two clusters with an immature signature

(A) Monocle 3 trajectory projected on the UMAP. Monocle 3 trajectory analysis and box plot representation of clusters cells across pseudotime with clusters reordered from the least to the highest mean pseudotime on **(B)** Colon tissue only, **(C)** CRC only, and **(D)** PM only. Box plots show median (center line), 25th–75th percentiles (box), and whiskers extending to ±1.5×IQR; outliers plotted as individual points. Source data are provided as a Source Data file. **(E)** Velocity confidence score plotted on the UMAP. Data are from a total of 19 tissue samples from 11 patients analyzed in 11 independent experiments (one patient per experiment). nILC = naive Innate Lymphoid Cells, ieILC1 = intraepithelial ILC1, NK = Natural Killer, CRC = Colorectal Cancer, PM = Peritoneal Metastasis.

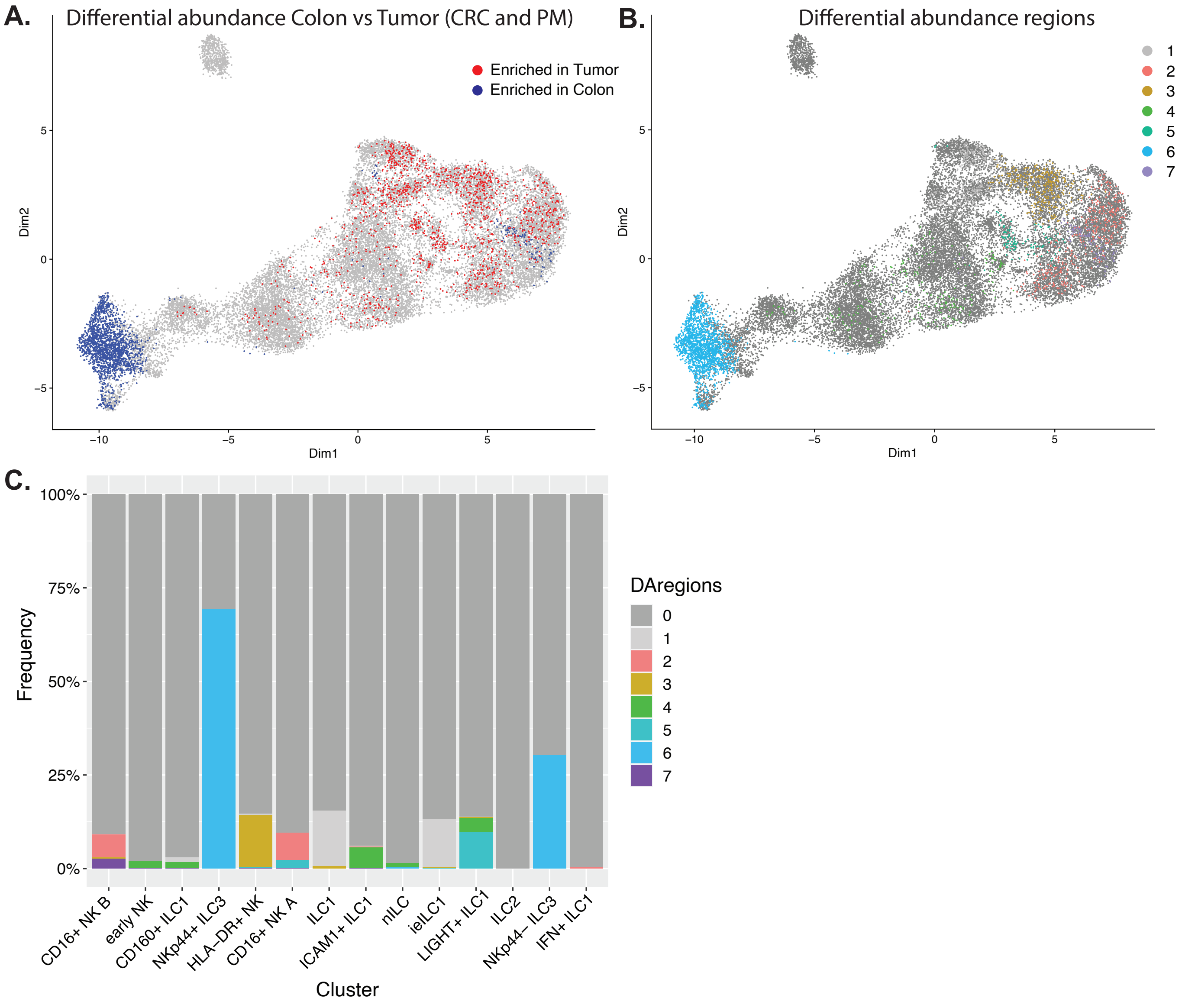

Supplementary Figure 3. ILC cluster compositions between unaffected colon, primary CRC and PM tumors

(A) Differentially abundant cells identified with DAseq and visualized in UMAP. Cells differentially abundant in colon are displayed in blue and in CRC and PM tumors in red. **(B)** Clusters of differentially abundant cells regrouped in cell regions and visualized by UMAP **(C)** Frequency of differentially abundant cell regions among the different scRNAseq clusters. Source data are provided as a Source Data file. Data are from a total of 19 tissue samples from 11 patients analyzed in 11 independent experiments (one patient per experiment). nILC = naive Innate Lymphoid Cells, ieILC1 = intraepithelial ILC1, NK = Natural Killer.

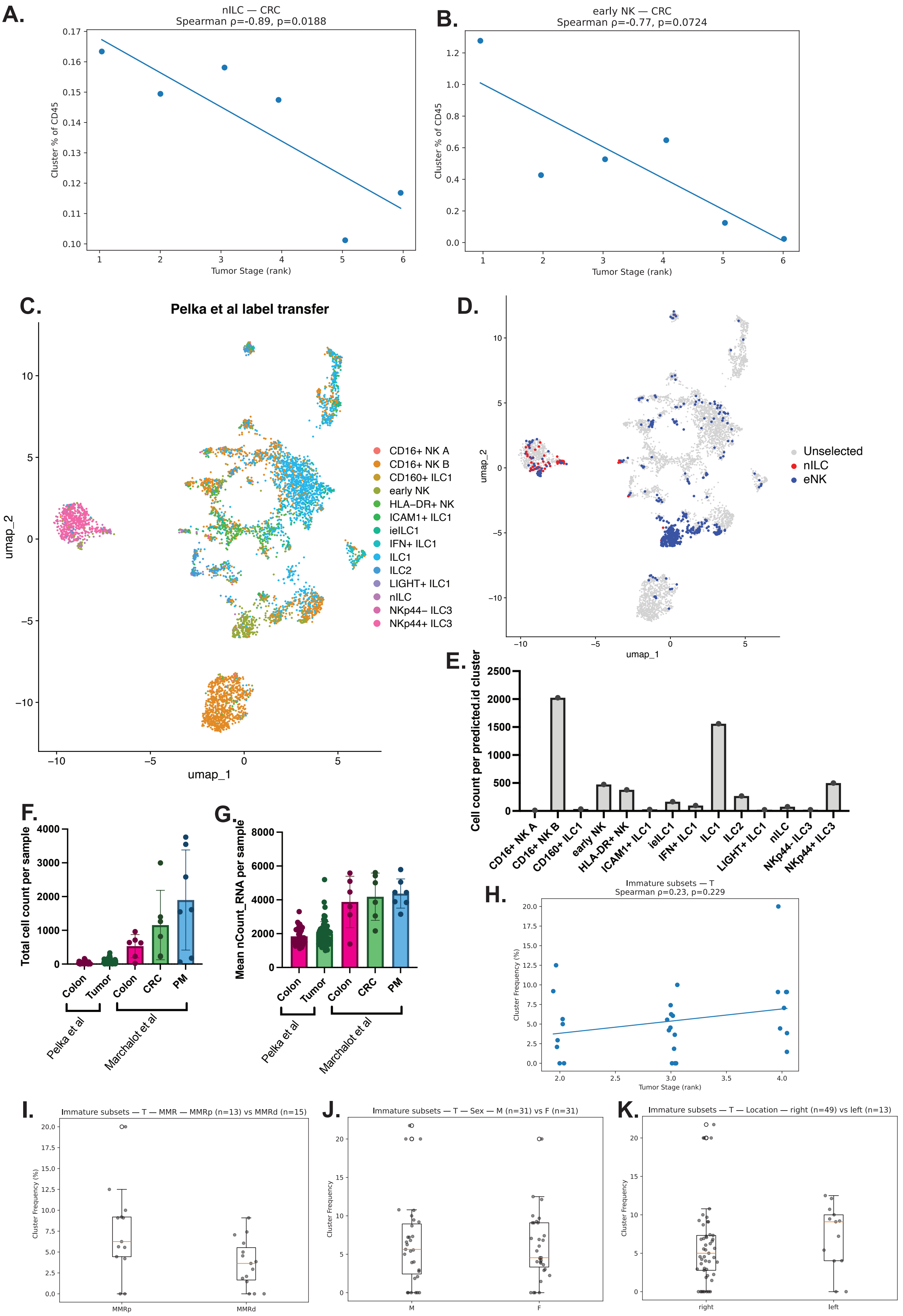
A.
nILC — CRC
Spearman ρ=-0.89, p=0.0188
Cluster % of CD45
Tumor Stage (rank)
B.
early NK — CRC
Spearman ρ=-0.77, p=0.0724
Cluster % of CD45
Tumor Stage (rank)
C.
Pelka et al label transfer
umap_1
umap_2
CD16+ NK A
CD16+ NK B
CD160+ ILC1
early NK
HLA-DR+ NK
ICAM1+ ILC1
ieILC1
IFN+ ILC1
ILC1
ILC2
LIGHT+ ILC1
nILC
NKp44- ILC3
NKp44+ ILC3
D.
umap_1
umap_2
Unselected
nILC
eNK
E.
Cell count per predicted.id cluster
F.
Total cell count per sample
Colon
Tumor
Colon
CRC
PM
Pelka et al
Marchalot et al
G.
Mean nCount_RNA per sample
Colon
Tumor
Colon
CRC
PM
Pelka et al
Marchalot et al
H.
Immature subsets — T
Spearman ρ=0.23, p=0.229
Cluster Frequency (%)
Tumor Stage (rank)
I.
Immature subsets — T — MMR — MMRp (n=13) vs MMRd (n=15)
Cluster Frequency (%)
MMRp
MMRd
J.
Immature subsets — T — Sex — M (n=31) vs F (n=31)
Cluster Frequency
M
F
K.
Immature subsets — T — Location — right (n=49) vs left (n=13)
Cluster Frequency
right
left

Supplementary Figure 4. eNK and nILC can be identified in Pelka et al scRNAseq dataset and correlated with tumor staging

(A) Correlation of nILC and **(B)** eNK as percentage of $CD45^+$ cells (as assessed by flow cytometry during FACS) with tumor stage rank. Spearman rank correlation with Benjamini–Hochberg correction. **(C)** Cluster label transfer on ILC and NK cells from Pelka et al scRNAseq dataset (GSE174381) visualized by UMAP. **(D)** Cells corresponding to nILC and eNK cells (by label transfer) highlighted on the UMAP in C. **(E)** Bar plot of cell counts for each label transferred cluster in the Pelka et al dataset. **(F)** Total cell count per sample and **(G)** mean RNA count per sample in Pelka et al. dataset compared to present dataset. Colon (magenta), CRC tumor (green) and PM (blue) **(H)** Correlation of tumor immature subsets (nILCs plus eNK cells) with tumor stage rank. Spearman rank correlation with Benjamini–Hochberg correction. Correlation of tumor immature subsets (nILCs plus eNK cells) with **(I)** MMR status, **(J)** sex and **(K)** tumor location. Two-sided Mann–Whitney U test with Benjamini–Hochberg correction. Source data are provided as a Source Data file. nILC = naive Innate Lymphoid Cells, ieILC1 = intraepithelial ILC1, eNK = early Natural Killer, MMRp/d = MisMatch Repair proficient/deficient.

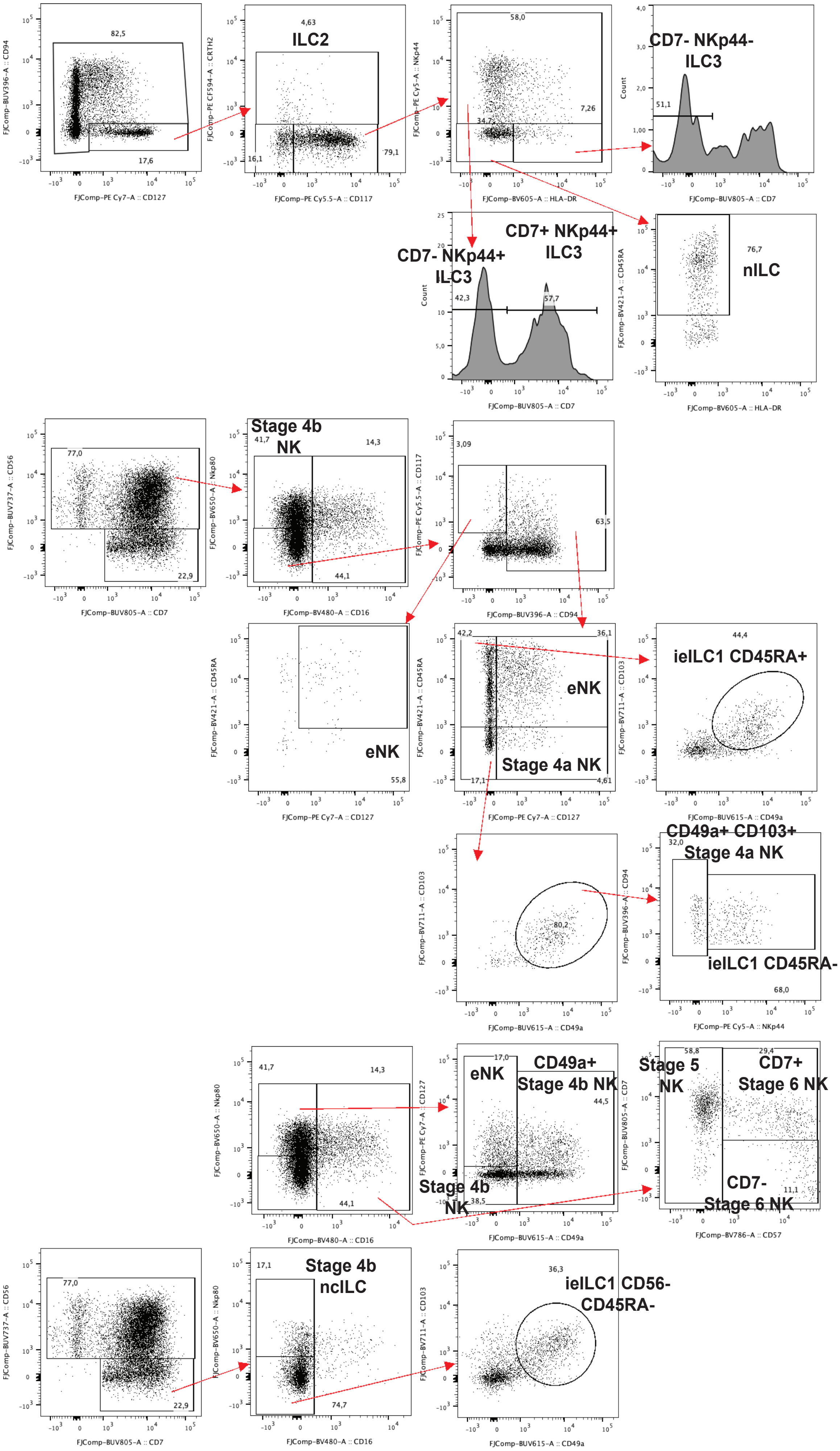

FJComp-BUV396-A :: CD94
FJComp-PE Cy7-A :: CD127
82,5
17,6
FJComp-PE CF594-A :: CRTH2
FJComp-PE Cy5.5-A :: CD117
4,63
ILC2
16,1
79,1
FJComp-PE Cy5-A :: NKp44
FJComp-BV605-A :: HLA-DR
58,0
7,26
34,7
Count
FJComp-BUV805-A :: CD7
CD7- NKp44-
ILC3
51,1
CD7+ NKp44+
ILC3
CD7- NKp44+
ILC3
42,3
57,7
FJComp-BV421-A :: CD45RA
76,7
nILC
FJComp-BUV737-A :: CD56
77,0
22,9
FJComp-BV650-A :: Nkp80
FJComp-BV480-A :: CD16
Stage 4b
NK
41,7
14,3
44,1
FJComp-PE Cy5.5-A :: CD117
3,09
63,5
eNK
55,8
42,2
36,1
eNK
Stage 4a NK
17,1
4,61
FJComp-BV711-A :: CD103
FJComp-BUV615-A :: CD49a
44,4
ielLC1 CD45RA+
80,2
CD49a+ CD103+
Stage 4a NK
32,0
ielLC1 CD45RA-
68,0
FJComp-PE Cy5-A :: NKp44
CD49a+
Stage 4b NK
eNK
17,0
44,5
Stage 4b
NK
38,5
Stage 5
NK
58,8
CD7+
Stage 6 NK
29,4
CD7-
Stage 6 NK
11,1
FJComp-BV786-A :: CD57
Stage 4b
ncILC
17,1
74,7
36,3
ielLC1 CD56-
CD45RA-

Supplementary Figure 5. Manual gating of flow cytometry data of total ILC and NK cells in the UMAP of Fig 5. nILC = naive Innate Lymphoid Cells, ieILC1 = intraepithelial ILC1, ncILC = non conventional ILC, eNK = early Natural Killer.

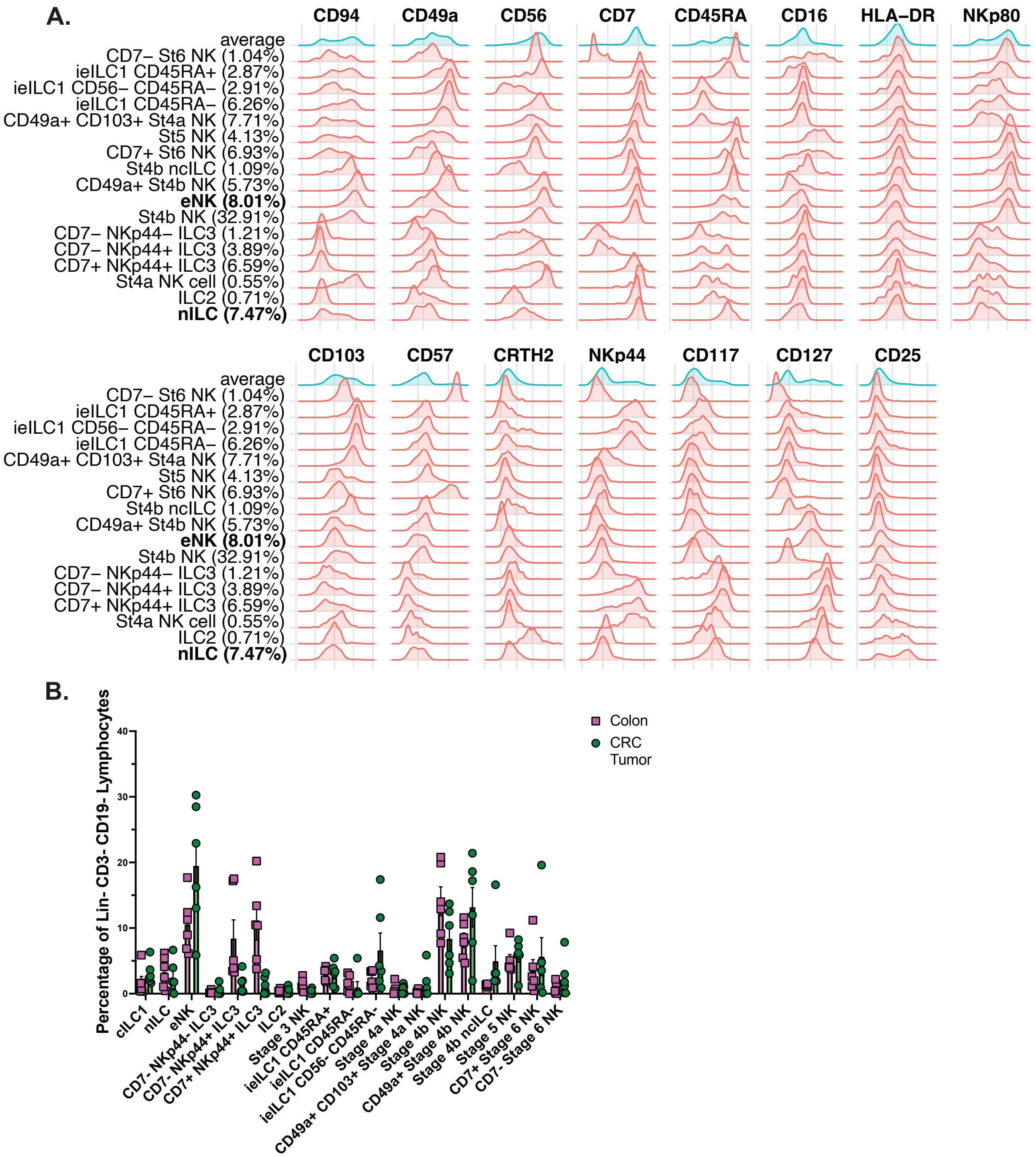

Supplementary Figure 6. Flow cytometric characterization of eNK cells and nILCs in unaffected colon and primary CRC tumors

(A) Expression of surface protein markers on ILC and NK cell clusters as defined in main figure 4C-D. **(B)** Bar plot of ILC and NK cells populations in Colon (magenta square) and CRC tumor (green circle) as characterized by manual gating of the clusters defined in the UMAP of main Figure 5. Source data are provided as a Source Data file. Data are from a total of 12 tissue samples from 7 patients analyzed in 2 independent experiments (3-4 patients per experiment). Multiple unpaired t-test (two sided) with Holm-Šídák correction, mean ± SEM. nILC = naive Innate Lymphoid Cells, ieILC1 = intraepithelial ILC1, ncILC = non conventional ILC, eNK = early Natural Killer, CRC = Colorectal Cancer.

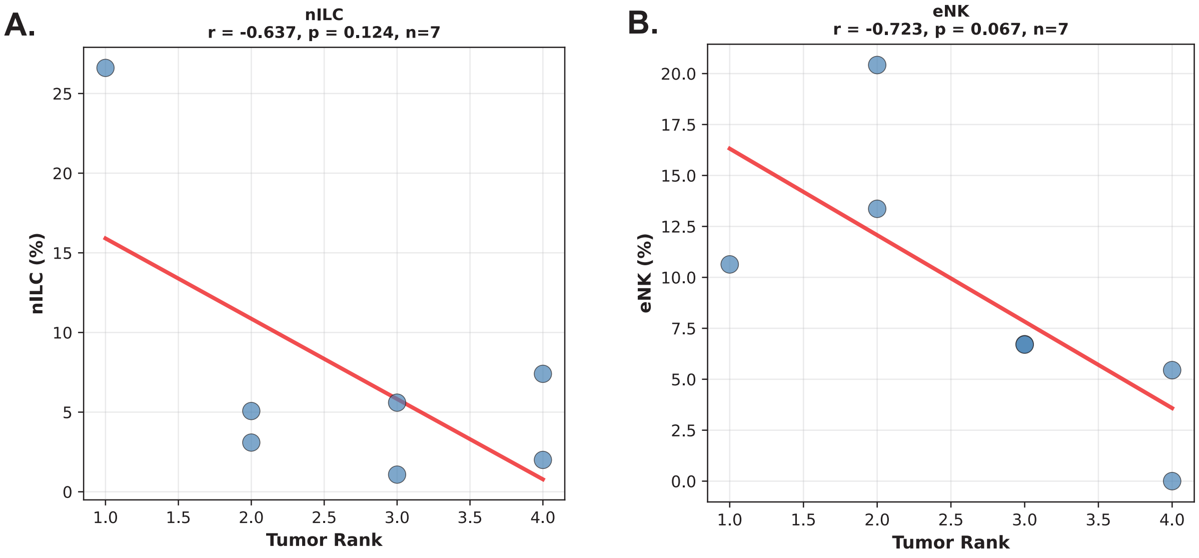

Supplementary Figure 7. Correlation of nILC and eNK cell clusters identified by flow cytometry with tumor stage

Correlation of nILC **(A)** and eNK **(B)** cell clusters identified by flow cytometry with tumor stage rank. Spearman rank correlation with Benjamini–Hochberg correction. Source data are provided as a Source Data file. nILC = naive Innate Lymphoid Cells, eNK = early Natural Killer.

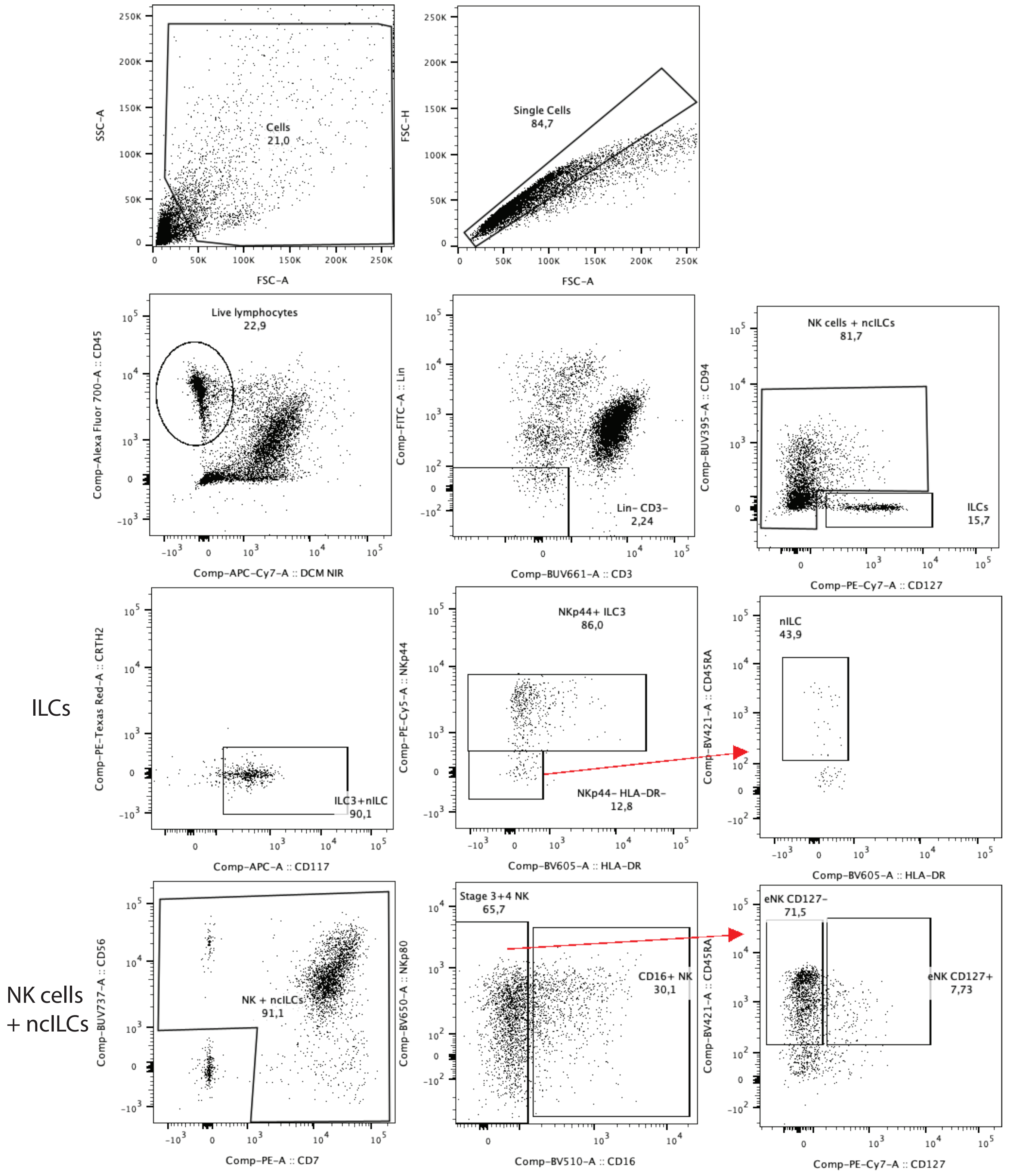

SSC-A
FSC-A
Cells
21,0
FSC-H
Single Cells
84,7
Comp-Alexa Fluor 700-A :: CD45
Comp-APC-Cy7-A :: DCM NIR
Live lymphocytes
22,9
Comp-FITC-A :: Lin
Comp-BUV661-A :: CD3
Lin- CD3-
2,24
Comp-BUV395-A :: CD94
Comp-PE-Cy7-A :: CD127
NK cells + ncILCs
81,7
ILCs
15,7
ILCs
Comp-PE-Texas Red-A :: CRTH2
Comp-APC-A :: CD117
ILC3+nILC
90,1
Comp-PE-Cy5-A :: NKp44
Comp-BV605-A :: HLA-DR
NKp44+ ILC3
86,0
NKp44- HLA-DR-
12,8
Comp-BV421-A :: CD45RA
Comp-BV605-A :: HLA-DR
nILC
43,9
NK cells
+ ncILCs
Comp-BUV737-A :: CD56
Comp-PE-A :: CD7
NK + ncILCs
91,1
Comp-BV650-A :: NKp80
Comp-BV510-A :: CD16
Stage 3+4 NK
65,7
CD16+ NK
30,1
Comp-BV421-A :: CD45RA
Comp-PE-Cy7-A :: CD127
eNK CD127-
71,5
eNK CD127+
7,73

Supplementary Figure 8. Gating strategy for sorting of nILCs and eNK cells from unaffected colon and primary CRC tumors for OP9-DL1 co-culture presented on Fig. 6, and Supplementary Fig. 10 and 11

Data are from a representative donor of a total of 14 tissue samples from 7 patients analyzed in 4 independent experiments (1-2 patients per experiment). nILC = naive Innate Lymphoid Cells, ncILC = non conventional ILC, eNK = early Natural Killer.

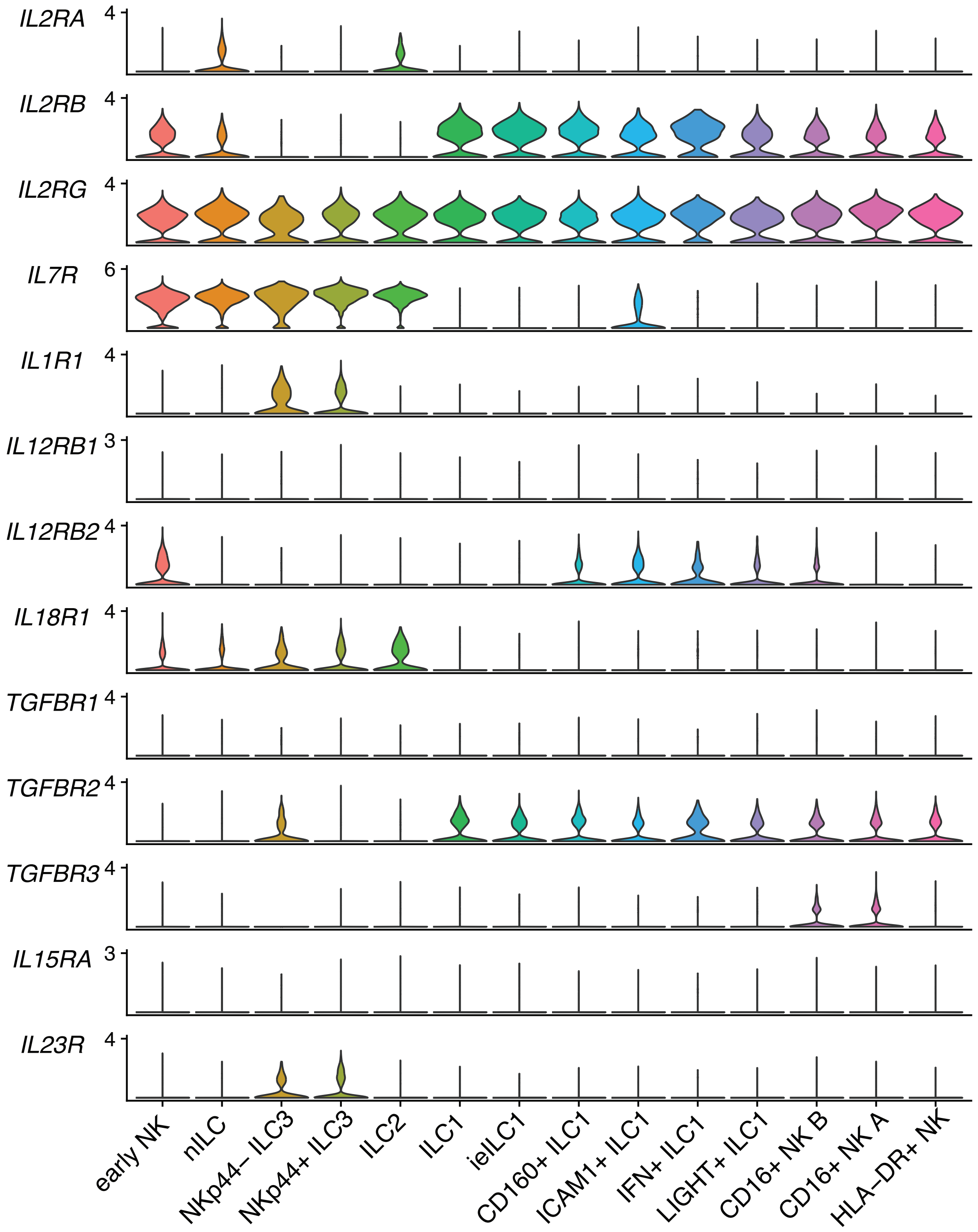

Supplementary Figure 9. Cytokine receptor expression in ILC and NK cell clusters

Data are from a total of 19 tissue samples from 11 patients analyzed in 11 independent experiments (one patient per experiment). nILC = naive Innate Lymphoid Cells, ieILC1 = intraepithelial ILC1, eNK = early Natural Killer.

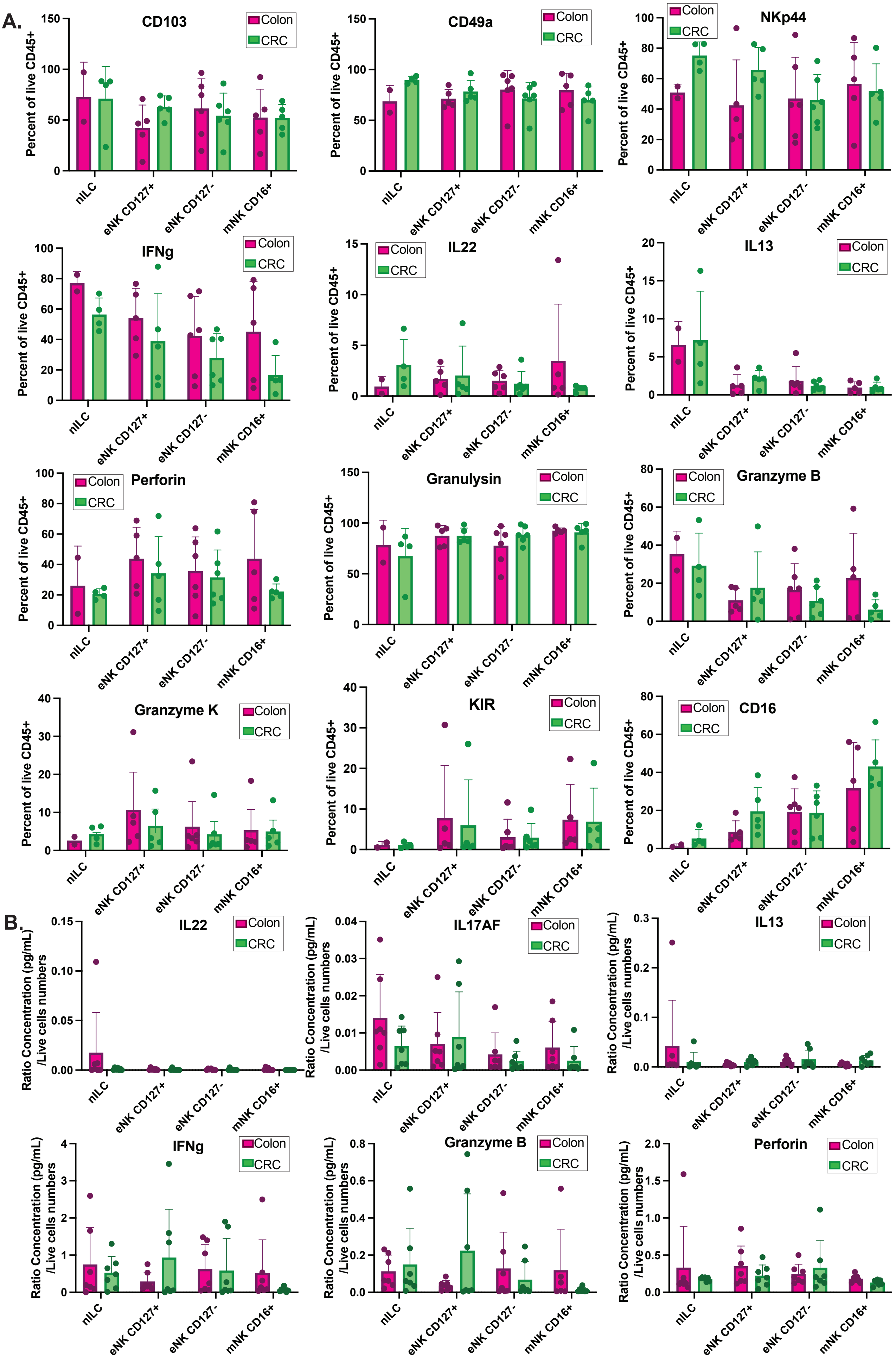
A.
CD103
CD49a
NKp44
IFNg
IL22
IL13
Perforin
Granulysin
Granzyme B
Granzyme K
KIR
CD16
Percent of live CD45+
Colon
CRC
nILC
eNK CD127+
eNK CD127-
mNK CD16+
B.
IL22
IL17AF
IL13
IFNg
Granzyme B
Perforin
Ratio Concentration (pg/mL)
/Live cells numbers

Supplementary Figure 10. Differentiation capacity of colon and intratumoral nILC in the OP9-DL1 system

nILCs, $CD127^+$ and $CD127^-$ eNK cells and $CD16^+$ NK cells from paired colon (magenta) and CRC (green) samples were cocultured for 14 days in presence of OP9-DL1 cells and IL-7, IL-2, IL-12, IL-18, IL-1β and TGF-β **(A)** Flow cytometry bar plots of proteins measured at the end of the culture (mean with standard deviation) **(B)** Concentrations of different proteins present in supernatant at the end of the culture as measured by bead-based multiplex immunoassays. Source data are provided as a Source Data file. Data are from a total of 14 tissue samples from 7 patients analyzed in 4 independent experiments (1-2 patients per experiment for flow cytometry, all samples analyzed in one bead-based multiplex immunoassay). Multiple paired t-test (two sided) with Holm-Šídák correction, mean ± SEM, revealed no significant differences between colon and CRC tumor samples. nILC = naive Innate Lymphoid Cells, eNK = early Natural Killer, CRC = Colorectal Cancer.

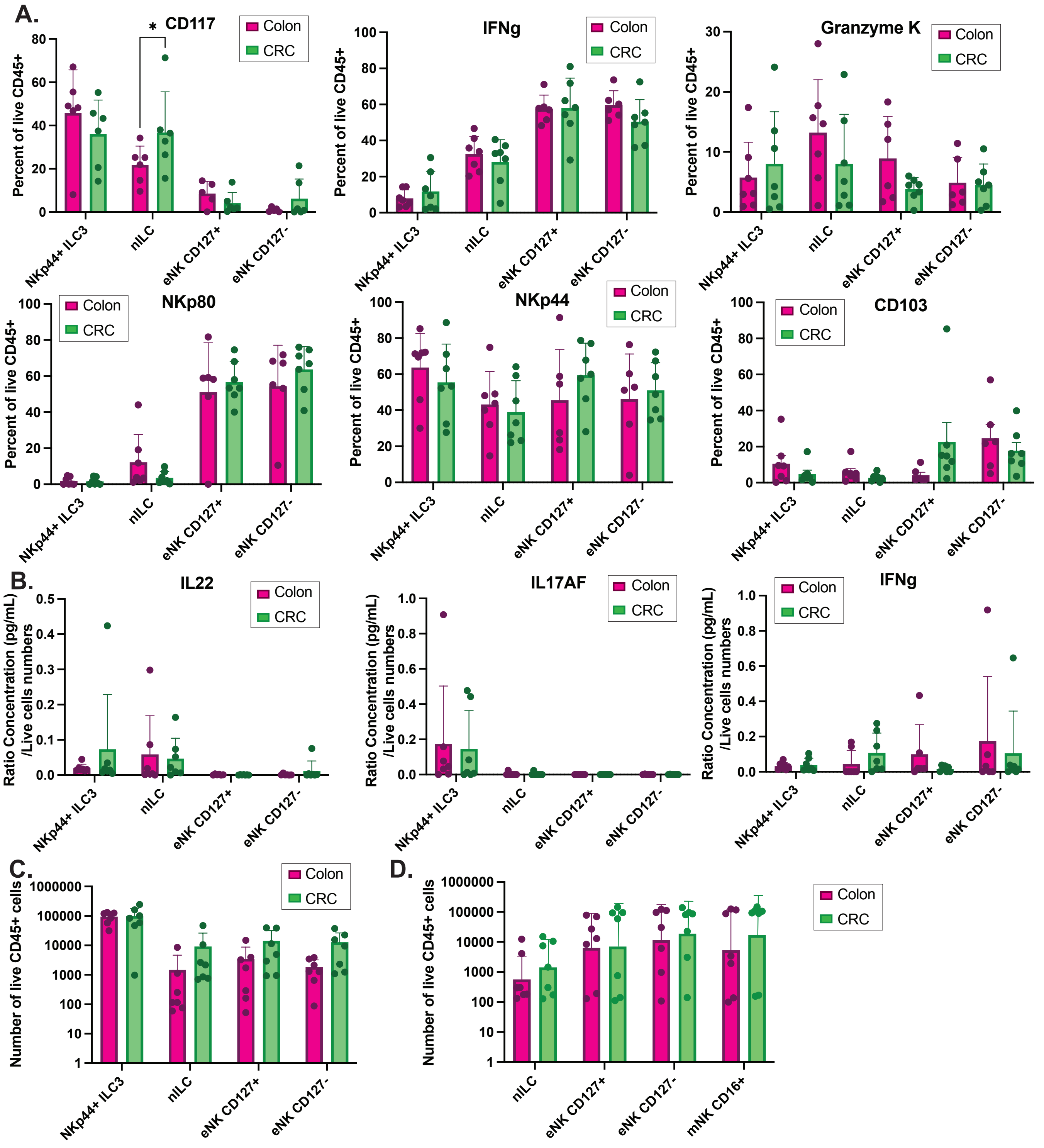
A.
CD117
*
Colon
CRC
Percent of live CD45+
0
20
40
60
80
NKp44+ ILC3
nILC
eNK CD127+
eNK CD127-
IFNg
Colon
CRC
Percent of live CD45+
0
20
40
60
80
100
NKp44+ ILC3
nILC
eNK CD127+
eNK CD127-
Granzyme K
Colon
CRC
Percent of live CD45+
0
10
20
30
NKp44+ ILC3
nILC
eNK CD127+
eNK CD127-
NKp80
Colon
CRC
Percent of live CD45+
0
20
40
60
80
100
NKp44+ ILC3
nILC
eNK CD127+
eNK CD127-
NKp44
Colon
CRC
Percent of live CD45+
0
20
40
60
80
100
NKp44+ ILC3
nILC
eNK CD127+
eNK CD127-
CD103
Colon
CRC
Percent of live CD45+
0
20
40
60
80
100
NKp44+ ILC3
nILC
eNK CD127+
eNK CD127-
B.
IL22
Colon
CRC
Ratio Concentration (pg/mL) /Live cells numbers
0.0
0.1
0.2
0.3
0.4
0.5
NKp44+ ILC3
nILC
eNK CD127+
eNK CD127-
IL17AF
Colon
CRC
Ratio Concentration (pg/mL) /Live cells numbers
0.0
0.2
0.4
0.6
0.8
1.0
NKp44+ ILC3
nILC
eNK CD127+
eNK CD127-
IFNg
Colon
CRC
Ratio Concentration (pg/mL) /Live cells numbers
0.0
0.2
0.4
0.6
0.8
1.0
NKp44+ ILC3
nILC
eNK CD127+
eNK CD127-
C.
Colon
CRC
Number of live CD45+ cells
1
10
100
1000
10000
100000
1000000
NKp44+ ILC3
nILC
eNK CD127+
eNK CD127-
D.
Colon
CRC
Number of live CD45+ cells
1
10
100
1000
10000
100000
1000000
nILC
eNK CD127+
eNK CD127-
mNK CD16+

Supplementary Figure 11. Differentiation capacity of colon and intratumoral nILC in the OP9-DL1 system

(A) Bar plots showing protein expression as measured by flow cytometry among $CD45^+$ live cells after 14 days of OP9-DL1 coculture with OP9-DL1 and IL-2, IL-7, IL-1β plus IL-23. Colon in magenta and CRC in green. **(B)** Calculated ratio of supernatant concentration of cytokines as measured by bead-based multiplex immunoassays divided by the number of live $CD45^+$ cells acquired by flow cytometry. **(C)** Number of live CD45+ cells as acquired by flow cytometry after 14 days of OP9-DL1 coculture with OP9-DL1 and IL-2, IL-7, IL-1β plus IL-2. **(D)** Number of live CD45+ cells as acquired by flow cytometry after 14 days in presence of OP9-DL1 cells and IL-7, IL-2, IL-12, IL-18, IL-1β and TGF-β. Source data are provided as a Source Data file. Data are from a total of 14 tissue samples from 7 patients analyzed in 4 independent experiments (1-2 patients per experiment for flow cytometry, all samples analyzed in one bead-based multiplex immunoassay). Multiple paired t-test (two sided) with Holm-Šídák correction, mean ± SEM, revealed no significant differences between colon and CRC tumor samples. nILC = naive Innate Lymphoid Cells, eNK = early Natural Killer, CRC = Colorectal Cancer.

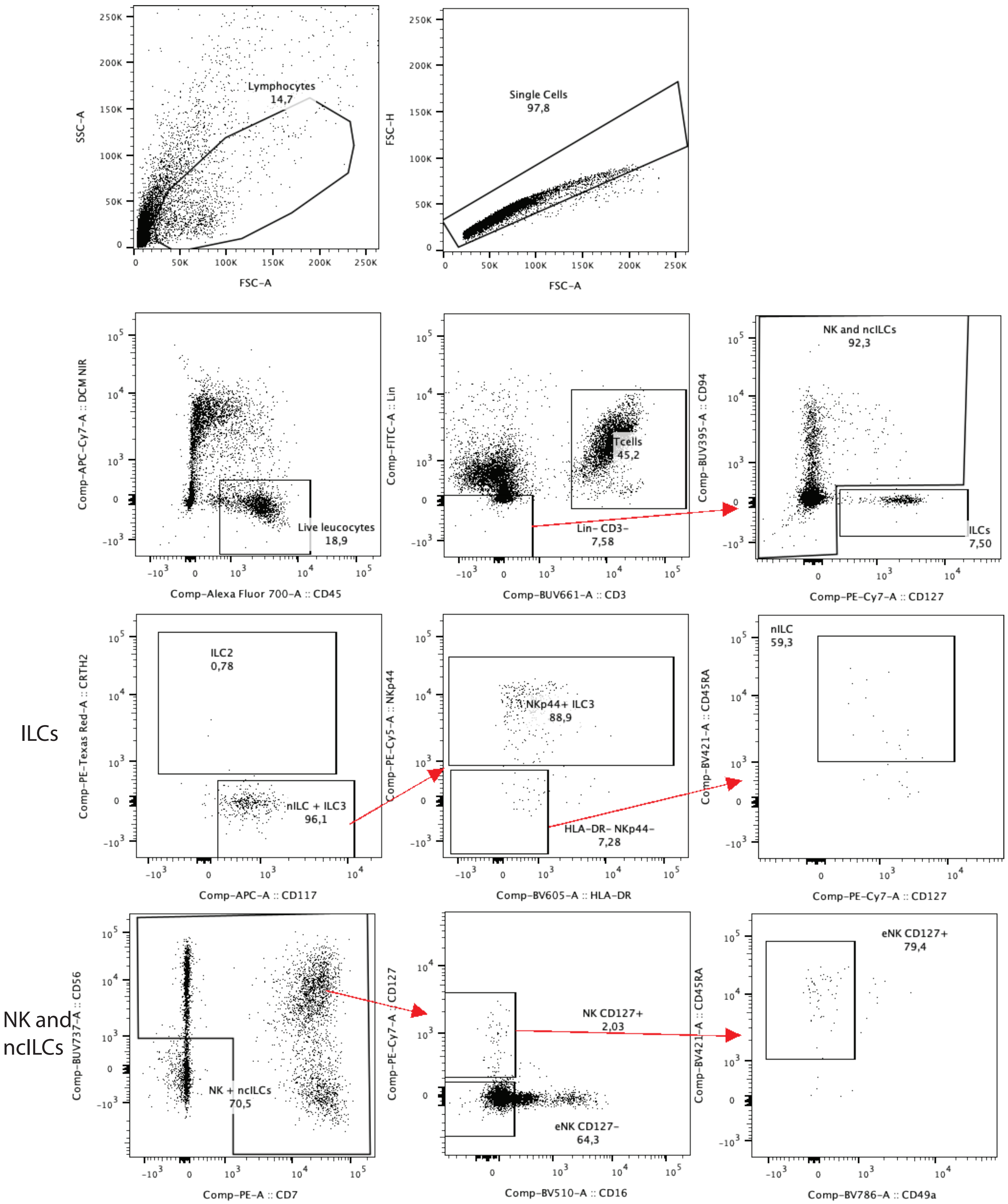

Supplementary Figure 12. Gating strategy for sorting of nILCs and eNK cells from unaffected colon and primary CRC tumors for Caco-2 co-cultures presented on Fig. 7 and S13. nILC = naive Innate Lymphoid Cells, ncILC = non conventional ILC, eNK = early Natural Killer, CRC = Colorectal Cancer.

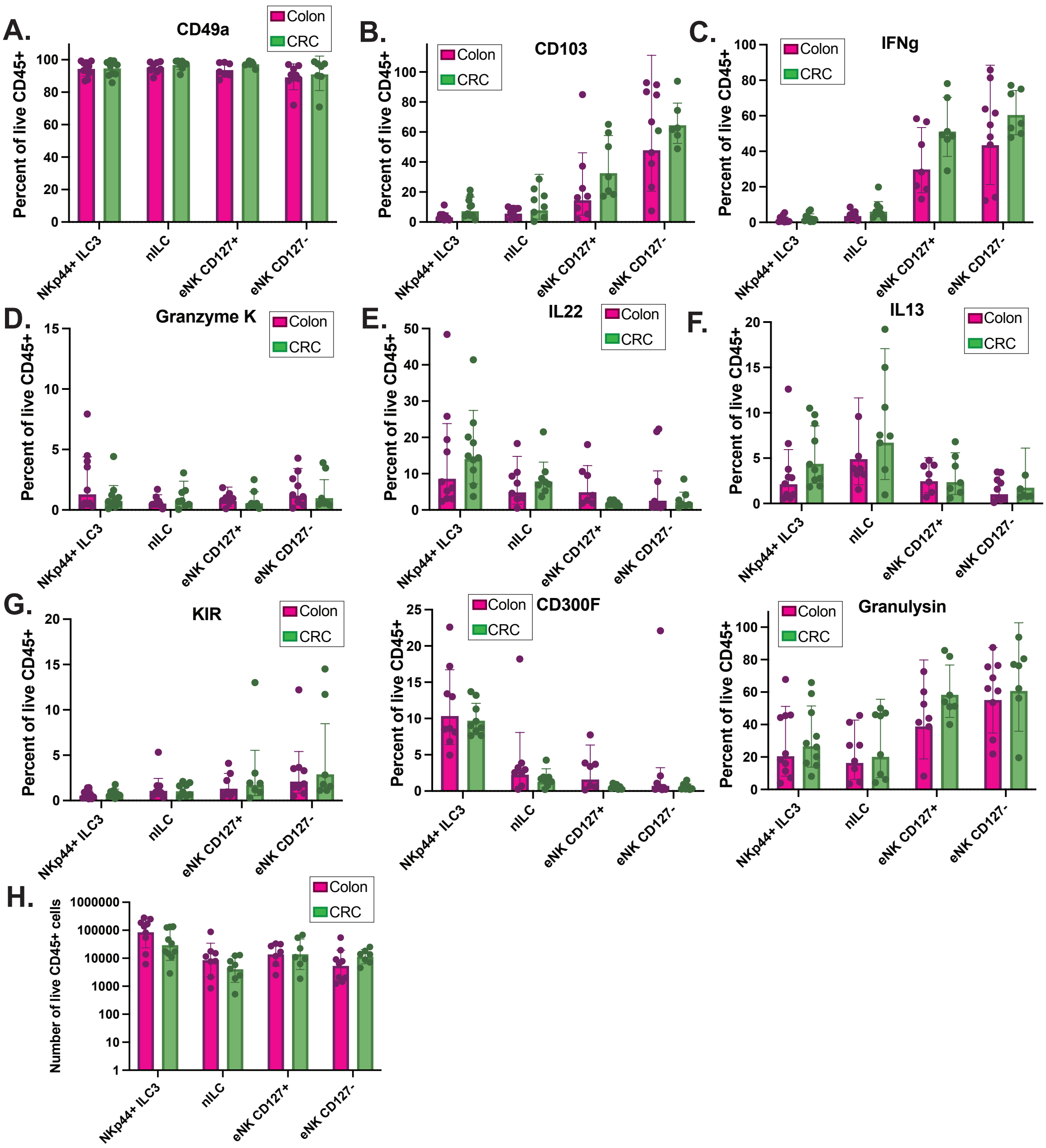

Supplementary Figure 13. Differentiation capacity of colon and intratumoral nILC in Caco-2 co-cultures

Bar plots showing protein expression (mean with standard deviation) as measured by flow cytometry among $CD45^+$ live cells after 10 days of Caco-2 coculture and IL-2, IL-7, IL-1β , IL-23 and IL-15. **(A)** CD49a **(B)** CD103 **(C)** Interferon gamma **(D)** Granzyme K **(E)** IL-22 **(F)** IL-13 **(G)** KIR, CD300F and Granulysin. **(H)** Bar plots showing the number of live $CD45^+$ cells after 10 days of coculture. Colon in magenta and CRC tumor in green. Source data are provided as a Source Data file. Data are from a total of 20 tissue samples from 10 patients analyzed in 5 independent experiments (2 patients per experiment). Multiple paired t-test (two sided) with Holm-Šídák correction, mean ± SEM, revealed no significant differences between colon and CRC tumor samples. nILC = naive Innate Lymphoid Cells, eNK = early Natural Killer, CRC = Colorectal Cancer.

Supplementary Table 1. Antibodies & reagents reference list

Antibody/Reagent name	Manufacturer	Product number	RRID	Dilution (Antibodies)
CD1a FITC	Biolegend	300104	AB_314018	2/50
CD14 FITC	LifeTechnologies	MHCD14014	AB_2539730	2/50
CD34 FITC	Biolegend	343504	AB_1731852	2/50
CD123 FITC	Biolegend	306014	AB_2124259	2/50
BCDA2 FITC	Miltenyi	130-113-754	AB_2726294	2/50
FCeR1a FITC	Biolegend	334608	AB_1227653	2/50
TCR ab FITC	Biolegend	306706	AB_314644	2/50
TCR gd FITC	Biolegend	331208	AB_1575108	2/50
CD4 FITC	Biolegend	317408	AB_571951	2/50
CD8a FITC	BD Biosciences	557085	AB_396580	2/50
CD19 FITC	BD Biosciences	555412	AB_395812	2/50
Granzyme B FITC	BD Biosciences	561998	AB_1645488	15/50
Granzyme K PerCP-eFluor710	Thermo Fisher	G3H69	AB_2573854	1/50
IREM-1 (CD300F) RB744	BD Biosciences	757132	AB_3689297	1,5/50
NKG2A APC	Beckman Coulter	A60797	AB_10643105	3/50
LIGHT APC	R&D Systems	FAB664A	AB_2206957	5/50
CD117 APC	Beckman Coulter	B36300	NA	5/50
IL13 APC	BD Biosciences	561162	AB_10642586	1/50
CD45 AF700	Biolegend	304024	AB_493761	2/50
CD62L AF700	Biolegend	304820	AB_493769	3/50
CD45RA BV421	BD Biosciences	562885	AB_2737864	2/50
IFNg BV421	BD Biosciences	562988	AB_2737934	1/50
Strepavidin BV510	Biolegend	405234	NA	1/50
CD16 V500	BD Biosciences	561393	AB_10611857	2/50
CD45 BV570	Biolegend	304033	AB_10899568	2/50
CD90 BV605	Biolegend	328128	AB_2562281	2/50
HLA-DR BV605	Biolegend	307640	AB_2561913	2/50
CD56 BV650	Biolegend	318344	AB_2563838	3/50
Strepavidin BV650	Biolegend	405231	NA	0,5/50
CD3 BV711	Biolegend	344838	AB_2565827	2/50
CD103 BV711	BD Biosciences	563162	AB_2738039	2/50
Granulysin BV750	BD Biosciences	624380	Custom antibody clone RB1	0,5/50
CD19 BV786	BD Biosciences	740968	AB_3685242	3/50
CD57 BV785	Biolegend	393330	AB_2860968	1/50
CD7 PE	Biolegend	395604	AB_2820049	2/50
CD5 PE	Biolegend	300608	AB_314094	2/50
IL22 PE	Biolegend	366703	AB_2565568	1/50
CD25 PE	Biolegend	302606	AB_314276	3/50
CRTH2 PE-CF594	BD Biosciences	563501	AB_2738244	2/50
CD127 (IL-7Ra) PE-Cy5	Biolegend	351324	AB_10915554	3/50
NKp44 (CD336) PC5	Beckman Coulter	A66903	NA	3/50
CD158a (KIR) PE-Cy5.5	Beckman Coulter	A66898	AB_2857330	2,5/50
CD117 PC5.5	Beckman Coulter	B96754	NA	3/50
CD38 PE-Cy7	Biolegend	303516	AB_2072782	2/50
CD127 PE-Cy7	Beckman Coulter	A64618	AB_2833031	3/50
Perforin PE-Cy7	Biolegend	353316	AB_2571973	0,5/50
CD94 BUV395	BD Biosciences	743954	AB_2741876	3/50
CD45 BUV395	BD Biosciences	563792	AB_2869519	1/50
CD11c BUV496	BD Biosciences	741139	AB_2916917	5/50
CD49a BUV615	BD Biosciences	751437	AB_2875433	0,5/50
CD3 BUV661	BD Biosciences	741692	AB_3685321	2/50
CD56 BUV737	BD Biosciences	612766	AB_2813880	1/50
CD7 BUV805	BD Biosciences	753775	AB_3687448	2/50
CD49a BV786	BD Biosciences	742362	AB_2740720	2/50
CD103 BUV737	BD Biosciences	568350	AB_3676338	1/50
NKG2D BV711	Biolegend	320848	AB_3097224	5/50
NKp46 BV786	BD Biosciences	563329	AB_2738139	2/50
NKp80 biotin	Miltenyi	130-095-114	AB_2857761	2/50
CD16 biotin	BD Biosciences	555405	AB_395805	2/50
LIVE/DEAD™ Fixable Near-IR Dead Cell Stain Kit	Thermo Fisher	L10119	NA	1/50
HyClone™ DPBS	Cytiva	SH30028.02	NA	
Fetal Bovine Serum	Sigma-Aldrich	F7524	NA	
EDTA (0.5 M), pH 8.0	LifeTechnologies	AM9260G	NA	
IMDM Gibco	Thermo Fisher	12440053	NA	
Bovine Serum Albumin Fraction V	Sigma-Aldrich	10735094001	NA	
Insulin from bovine pancreas	Sigma-Aldrich	I5500	NA	
Ethanolamine	EMD Millipore	8008490500	NA	
Transferrin from human serum	Roche	10652202001	NA	
HyClone™ Penicillin-Streptomycin	Cytiva	SV30010	NA	
BD Cytofix/Cytoperm™ Fixation/Permeabilization Solution Kit	BD Biosciences	554714	AB_2869008	
BD GolgiPlug™ Protein Transport Inhibitor	BD Biosciences	555029	AB_2869014	
BD GolgiStop™ Protein Transport Inhibitor	BD Biosciences	554724	AB_2869012	
Phorbol 12-myristate 13-acetate (PMA)	Sigma-Aldrich	P8139	NA	
Ionomycin, Calcium Salt	Thermo Fisher	I24222	NA	
Collagenase from Clostridium histolyticum	Merk	C6885-100MG	NA	
DNase I	Merk	10104159001	NA	
DMSO	Merk	10104159001	NA	
