# Supplementary material for: Tumor-infiltrating immature innate lymphoid cells in colorectal cancer are biased toward ILC1/tissue-resident NK cell differentiation
Source: Nat Commun. 2026 Mar 27;17:3035. doi: 10.1038/s41467-026-71085-9 (PMC13035902; doi:10.1038/s41467-026-71085-9)
Supplement: Supplementary file 2 — Description of Additional Supplementary Files [file 41467_2026_71085_MOESM2_ESM.pdf]

Description of Additional Supplementary Files

Supplementary data 1 Differentially expressed genes identified between colon and colorectal cancer (CRC) naïve innate lymphoid cells (nILC). The table includes gene names, log2 fold change, percentage of expressing cells in colon (pct.1) and CRC (pct.2), and associated p values.

Supplementary data 2 Differentially expressed genes identified between colon and colorectal cancer (CRC) early Natural Killer cells (eNK). The table includes gene names, log2 fold change, percentage of expressing cells in colon (pct.1) and CRC (pct.2), and associated p values.
