# Supplementary material for: Tumor-infiltrating immature innate lymphoid cells in colorectal cancer are biased toward ILC1/tissue-resident NK cell differentiation
Source: Nat Commun. 2026 Mar 27;17:3035. doi: 10.1038/s41467-026-71085-9 (PMC13035902; doi:10.1038/s41467-026-71085-9)
Supplement: Supplementary file 5 — Reporting Summary [file 41467_2026_71085_MOESM5_ESM.pdf]

nature portfolio

Corresponding author(s): Jenny Mjösberg

Last updated by author(s): 12/02/2026

Reporting Summary

Nature Portfolio wishes to improve the reproducibility of the work that we publish. This form provides structure for consistency and transparency in reporting. For further information on Nature Portfolio policies, see our Editorial Policies and the Editorial Policy Checklist.

Statistics

For all statistical analyses, confirm that the following items are present in the figure legend, table legend, main text, or Methods section.

n/a	Confirmed	
☐	☒	The exact sample size (*n*) for each experimental group/condition, given as a discrete number and unit of measurement
☐	☒	A statement on whether measurements were taken from distinct samples or whether the same sample was measured repeatedly
☐	☒	The statistical test(s) used AND whether they are one- or two-sided<br>*Only common tests should be described solely by name; describe more complex techniques in the Methods section.*
☐	☒	A description of all covariates tested
☐	☒	A description of any assumptions or corrections, such as tests of normality and adjustment for multiple comparisons
☐	☒	A full description of the statistical parameters including central tendency (e.g. means) or other basic estimates (e.g. regression coefficient) AND variation (e.g. standard deviation) or associated estimates of uncertainty (e.g. confidence intervals)
☐	☒	For null hypothesis testing, the test statistic (e.g. *F*, *t*, *r*) with confidence intervals, effect sizes, degrees of freedom and *P* value noted<br>*Give P values as exact values whenever suitable.*
☒	☐	For Bayesian analysis, information on the choice of priors and Markov chain Monte Carlo settings
☒	☐	For hierarchical and complex designs, identification of the appropriate level for tests and full reporting of outcomes
☐	☒	Estimates of effect sizes (e.g. Cohen's *d*, Pearson's *r*), indicating how they were calculated

Our web collection on statistics for biologists contains articles on many of the points above.

Software and code

Policy information about availability of computer code

Data collection	*Provide a description of all commercial, open source and custom code used to collect the data in this study, specifying the version used OR state that no software was used.*
Data analysis	CellRanger version 7.0.1 (10X Genomics). R version 4.4.1 and packages : Seurat v4.3 and v5.3.1, Harmony v0.1.1 and v1.2.4, EnhancedVolcano v1.22, DASeq v1, scCustomize v2.1.2, scvelo v0.3.1, Monocle 3 package v1.3.7, CitoFWorkflow v4. Python version 3.12 and packages scvelo v0.3.1, Phenograph v1.5.2, Matplotlib v3.9 and v3.10.6, scipy v1.16.2, pandas v2.3.3 and numpy v2.3.3. FlowJo v5, Bio-Plex Manager, Adobe Illustrator 2023, GraphPad Prism 10, FACS Diva version 9. https://github.com/Mjosberg-Lab/ILC-NK-HIPEC

For manuscripts utilizing custom algorithms or software that are central to the research but not yet described in published literature, software must be made available to editors and reviewers. We strongly encourage code deposition in a community repository (e.g. GitHub). See the Nature Portfolio guidelines for submitting code & software for further information.

Data

Policy information about availability of data

All manuscripts must include a data availability statement. This statement should provide the following information, where applicable:

- Accession codes, unique identifiers, or web links for publicly available datasets
- A description of any restrictions on data availability
- For clinical datasets or third party data, please ensure that the statement adheres to our policy

The single cell RNA sequencing data generated in this study are available in the GEO database under accession code GSE302045. All data are included in the

Supplementary Information or available from the authors, as are unique reagents used in this Article. The raw numbers for charts and graphs are available in the Source Data file whenever possible. Pelka et al. scRNAseq CRC dataset is availabe under the accession code GSE178341.

Research involving human participants, their data, or biological material

Policy information about studies with human participants or human data. See also policy information about sex, gender (identity/presentation), and sexual orientation and race, ethnicity and racism.

Reporting on sex and gender	Analysis were performed regarding sex to unsure no statistical difference could be explained by a sex bias in the analysis. Sex was assigned according to the Swedish registry informations.
Reporting on race, ethnicity, or other socially relevant groupings	No data regarding race, ethnicity or other socially relevant grouping was collected for this study
Population characteristics	Age between 34 and 90
Recruitment	Patients undergoing elective resection for colorectal adenocarcinomas and/or colorectal PM at the colorectal cancer unit at Karolinska University Hospital were eligible for inclusion. Exclusions criteria were (i) neoadjuvant or adjuvant chemotherapy in the six months preceding the surgery, and (ii) inability to read and consent to study information. Participants were not compensated. All patients included gave informed written consent.
Ethics oversight	the swedish ethics review authority

Note that full information on the approval of the study protocol must also be provided in the manuscript.

Field-specific reporting

Please select the one below that is the best fit for your research. If you are not sure, read the appropriate sections before making your selection.

[x] Life sciences [ ] Behavioural & social sciences [ ] Ecological, evolutionary & environmental sciences

For a reference copy of the document with all sections, see nature.com/documents/nr-reporting-summary-flat.pdf

Life sciences study design

All studies must disclose on these points even when the disclosure is negative.

Sample size	scRNAseq sample size was determined by the limited available fresh samples at the time of the collection (2 years) and limited funding considering scRNAseq costs
Data exclusions	No data was excluded from the analyses when available
Replication	Reproductibility was assessed by repeating experiments with different patient samples and at different times
Randomization	Samples were allocated into experimental groups randomly by sample availability and processing time restrictions
Blinding	Blinding was not possible due to the nature of the samples and not necessary considering the small size of the experimental groups

Reporting for specific materials, systems and methods

We require information from authors about some types of materials, experimental systems and methods used in many studies. Here, indicate whether each material, system or method listed is relevant to your study. If you are not sure if a list item applies to your research, read the appropriate section before selecting a response.

Materials & experimental systems

n/a	Involved in the study	
[ ]	[x]	Antibodies
[ ]	[x]	Eukaryotic cell lines
[x]	[ ]	Palaeontology and archaeology
[x]	[ ]	Animals and other organisms
[ ]	[x]	Clinical data
[x]	[ ]	Dual use research of concern
[x]	[ ]	Plants

Methods

n/a	Involved in the study	
[x]	[ ]	ChIP-seq
[ ]	[x]	Flow cytometry
[x]	[ ]	MRI-based neuroimaging

Antibodies

Antibodies used	CD1a FITC Biolegend 300104<br>CD14 FITC LifeTechnologies MHCD14014<br>CD34 FITC Biolegend 343504<br>CD123 FITC Biolegend 306014<br>BCDA2 FITC Miltenyi 130-113-754<br>FCeR1a FITC Biolegend 334608<br>TCR ab FITC Biolegend 306706<br>TCR gd FITC Biolegend 331208<br>CD4 FITC Biolegend 317408<br>CD8a FITC BD Biosciences 557085<br>CD19 FITC BD Biosciences 555412<br>Granzyme B FITC BD Biosciences 561998<br>Granzyme K PerCP-eFluor710 Thermofisher G3H69<br>IREM-1 (CD300F) RB744 BD Biosciences 757132<br>NKG2A APC Beckman Coulter A60797<br>LIGHT APC R&D Systems FAB664A<br>CD117 APC Beckman Coulter B36300<br>IL13 APC BD Biosciences 561162<br>CD45 AF700 Biolegend 304024<br>CD62L AF700 Biolegend 304820<br>CD45RA BV421 BD Biosciences 562885<br>IFNg BV421 BD Biosciences 562988<br>Strepavidin BV510 Biolegend 405234<br>CD16 V500 BD Biosciences 561393<br>CD45 BV570 Biolegend 304033<br>CD90 BV605 Biolegend 328128<br>HLA-DR BV605 Biolegend 307640<br>CD56 BV650 Biolegend 318344<br>Strepavidin BV650 Biolegend 405231<br>CD3 BV711 Biolegend 344838<br>CD103 BV711 BD Biosciences 563162<br>Granulysin BV750 BD Biosciences 624380<br>CD19 BV786 BD Biosciences 740968<br>CD57 BV785 Biolegend 393330<br>CD7 PE Biolegend 395604<br>CD5 PE Biolegend 300608<br>IL22 PE Biolegend 366703<br>CD25 PE Biolegend 302606<br>CRTH2 PE-CF594 BD Biosciences 563501<br>CD127 (IL-7Ra) PE-Cy5 Biolegend 351324<br>NKp44 (CD336) PC5 Beckman Coulter A66903<br>CD158a (KIR) PE-Cy5.5 Beckman Coulter A66898<br>CD117 PC5.5 Beckman Coulter B96754<br>CD38 PE-Cy7 Biolegend 303516<br>CD127 PE-Cy7 Beckman Coulter A64618<br>Perforin PE-Cy7 Biolegend 353316<br>CD94 BUV395 BD Biosciences 743954<br>CD45 BUV395 BD Biosciences 563792<br>CD11c BUV496 BD Biosciences 741139<br>CD49a BUV615 BD Biosciences 751437<br>CD3 BUV661 BD Biosciences 741692<br>CD56 BUV737 BD Biosciences 612766<br>CD7 BUV805 BD Biosciences 753775<br>CD49a BV786 BD Biosciences 742362<br>CD103 BUV737 BD Biosciences 568350<br>NKG2D BV711 Biolegend 320848<br>NKp46 BV786 BD Biosciences 563329<br>NKp80 biotin Miltenyi 130-095-114<br>CD16 biotin BD Biosciences 555405
Validation	All the antibodies have been validated by the manufacturers : https://www.biolegend.com/sv-se, https://www.bdbiosciences.com/en-se, https://www.mybeckman.se/, https://www.rndsystems.com/, https://www.thermofisher.com/se/en/home.html,https://www.miltenyibiotec.com/DE-en/

Eukaryotic cell lines

Policy information about cell lines and Sex and Gender in Research

Cell line source(s)	OP9-DL1 by Hergens Spits Laboratory, Caco-2 cell lines by Peter Bergman laboratory
Authentication	Cells were authenticated and confirmed for Caco-2 by STR profiling (ATCC) on the 23/10/2025 but not OP9-DL1
Mycoplasma contamination	Cells were tested negative for mycoplasma
Commonly misidentified lines (See ICLAC register)	*Name any commonly misidentified cell lines used in the study and provide a rationale for their use.*

Clinical data

Policy information about clinical studies

All manuscripts should comply with the ICMJE guidelines for publication of clinical research and a completed CONSORT checklist must be included with all submissions.

Clinical trial registration	*Provide the trial registration number from ClinicalTrials.gov or an equivalent agency.*
Study protocol	*Note where the full trial protocol can be accessed OR if not available, explain why.*
Data collection	*Describe the settings and locales of data collection, noting the time periods of recruitment and data collection.*
Outcomes	*Describe how you pre-defined primary and secondary outcome measures and how you assessed these measures.*

Plants

Seed stocks	*Report on the source of all seed stocks or other plant material used. If applicable, state the seed stock centre and catalogue number. If plant specimens were collected from the field, describe the collection location, date and sampling procedures.*
Novel plant genotypes	*Describe the methods by which all novel plant genotypes were produced. This includes those generated by transgenic approaches, gene editing, chemical/radiation-based mutagenesis and hybridization. For transgenic lines, describe the transformation method, the number of independent lines analyzed and the generation upon which experiments were performed. For gene-edited lines, describe the editor used, the endogenous sequence targeted for editing, the targeting guide RNA sequence (if applicable) and how the editor was applied.*
Authentication	*Describe any authentication procedures for each seed stock used or novel genotype generated. Describe any experiments used to assess the effect of a mutation and, where applicable, how potential secondary effects (e.g. second site T-DNA insertions, mosiacism, off-target gene editing) were examined.*

Flow Cytometry

Plots

Confirm that:

- [x] The axis labels state the marker and fluorochrome used (e.g. CD4-FITC).
- [x] The axis scales are clearly visible. Include numbers along axes only for bottom left plot of group (a 'group' is an analysis of identical markers).
- [x] All plots are contour plots with outliers or pseudocolor plots.
- [x] A numerical value for number of cells or percentage (with statistics) is provided.

Methodology

Sample preparation	cells were first stained with dead cell marker (LIVE/DEAD Fixable Near-IR, Invitrogen) and a cocktail of surface antibodies for 20 to 30 min at 4°C. Antibodies reference list can be found in Table S3. For experiments involving intracellular staining (Granzymes, granulosin, perforin and interleukines) cells were fixed and permeabilized using the BD Cytofix/Cytoperm™ Fixation/Permeabilization Solution Kit. If no intracellular staining was needed, samples were fixed for 5min with PBS 2% paraformaldehyde buffer prior to acquisition.
Instrument	FACSymphony™ A3 or A5 (BD Biosciences)
Software	FlowJo version 10. R package CitoFWorkflow and python packages Matplotlib and Phenograph
Cell population abundance	Sorted cell population purity was assessed by taking a small sample of the sorted cells and analyzing them in the sorter instrument again. Purity was minimum 90%
Gating strategy	CD127+ ILCs (CD45+, CD3-, CD19-, Lin- and CD127+), NK cells (CD45+, CD3-, CD19-, Lin-, CD94+/- and CD56+) and non-conventional (nc)ILCs (CD45+, CD3-, CD19-, Lin-, CD94+/-, CD56- and CD7+), T cells (CD45+, CD3+) for scRNAseq cell sorting.

nILC (CD45+, Lin-CD127+CD94-CD117+CRTH2-NKp44-HLA-DR-CD45RA+) CD127+ eNK (CD45+, Lin-CD56+CD94+CD16-CD127lowCD45RA+) CD127- eNK (CD45+, Lin-CD56+CD94+CD16-CD127-), CD16+ NK (CD45+, Lin-CD56+CD94+CD16+), NKp44 + ILC3 (CD45+, Lin-CD127+CD94-CD117+CRTH2-NKp44+)

☒ Tick this box to confirm that a figure exemplifying the gating strategy is provided in the Supplementary Information.
